# Newly identified patterns of Pax2 expression in the developing mouse forebrain

**DOI:** 10.1186/1471-213X-8-79

**Published:** 2008-08-13

**Authors:** Vassiliki Fotaki, David J Price, John O Mason

**Affiliations:** 1 Genes and Development Group, Centres for Integrative Physiology and Neuroscience Research, School of Biomedical Sciences, University of Edinburgh, Hugh Robson Building, George Square, Edinburgh, EH8 9XD, UK

## Abstract

**Background:**

The availability of specific markers expressed in different regions of the developing nervous system provides a useful tool for the study of mouse mutants. One such marker, the transcription factor Pax2, is expressed at the midbrain-hindbrain boundary and in the cerebellum, spinal cord, retina, optic stalk, and optic chiasm. We recently described a group of diencephalic cells that express Pax2 as early as embryonic day (E) 10.5, and become part of the eminentia thalami by E11.5. The discovery of this previously undescribed cell population prompted us to examine Pax2 protein expression in the developing mouse forebrain in more detail.

**Results:**

We determined the expression pattern of Pax2 in the forebrain of wild type mouse embryos between E10.5 and postnatal day (P) 15. Pax2 expression was detected in the septum of the basal forebrain, hypothalamus, eminentia thalami and in the subfornical organ. To evaluate Pax2 as a marker for septal cells, we examined Pax2 expression in *Pax6*^*Sey*/*Sey *^mutants, which have an enlarged septum. We found that Pax2 clearly marks a population of septal cells equivalent to that seen in wild types, indicating its utility as a marker of septal identity. These cells did not express the GABAergic marker calbindin nor the cholinergic marker choline acetyltransferase and were not detectable after P15.

**Conclusion:**

Pax2 is expressed in populations of cells within the developing septum, hypothalamus, and eminentia thalami. It seems especially useful as a marker of the telencephalic septum, because of its early, strong and characteristic expression in this structure. Further, its expression is maintained in the enlarged septum of *Pax6*^*Sey*/*Sey *^mutants.

## Background

Pax2 is a member of the Pax family of transcription factors [1,2], characterised by the presence of a paired-type homeodomain [3,4]. *Pax2 *is expressed in a number of different organs in the developing mouse embryo, including the ureteric bud, kidneys [1,2] and otic vesicle [5]. In the developing nervous system, *Pax2 *is first detected at embryonic day (E) 7.5 in the neural plate, in the area of the presumptive midbrain-hindbrain region [6]. At E8.0, *Pax2 *displays a broad expression domain in this region, which by E9.5 is restricted to the isthmus at the midbrain-hindbrain boundary [6]. Pax2 expression in the isthmus ceases after E11 [7]. In the cerebellum, Pax2 is specifically expressed by a subset of cerebellar GABAergic interneurons and their precursors, from E12 until the end of cerebellar development (postnatal day 15) [8]. In the spinal cord, Pax2 expression is found in the intermediate zone as early as E10.5 [5,7]. In the developing eye,*Pax2 *expression is initiated at E9 in the ventral half of the optic vesicle. By E11, after invagination of the optic vesicle, both Pax2 transcript and protein are detected at high levels in the ventral opening of the optic cup, the optic fissure, and the optic stalk, ending at the border with the diencephalon [5,7,9,10]. After E12.5, Pax2 protein expression is still present in the ventral optic cup, although the level is decreased, and it is no longer detected after E16.5 [9]. Pax2 is also expressed in glial cells in the optic nerve [5,9,10].

Pax6, another member of the Pax family, is expressed in the dorsal telencephalon, diencephalon, hindbrain regions and spinal cord [11] and throughout the developing optic cup, but is absent from the optic stalk and optic nerve [12]. Mutant mice lacking functional Pax6 protein (*Pax6*^*Sey*/*Sey *^mice), display a large range of nervous system defects, including absence of eyes [13], disruption of dorsoventral telencephalic patterning [14,15] and the diencephalic-mesencephalic boundary [16,17]. Pax6 and Pax2 are expressed in neighbouring, but mutually exclusive domains in the developing eye (with the exception of the ventral optic cup), [18], and in the diencephalic-mesencephalic region [19]. In the developing spinal cord, Pax2 is expressed by many types of early differentiated neurons, located in the mantle zone and surrounding Pax6-positive neural precursors in the ventricular zone [20]. Fewer Pax2-positive interneurons are found in the *Pax6*^*Sey*/*Sey *^mutant, indicating that Pax6 is required for their development [20].

We have recently described a group of diencephalic cells at the border region between the diencephalon and the telencephalon that expresses Pax2 protein at E10.5 [21]. At E12.5, these Pax2-immunopositive cells form a distinct cell population at the most dorso-lateral tip of the eminentia thalami [21], a diencephalic structure that joins the ventral diencephalon to the dorsal and ventral telencephalon [22-24]. Here, we describe previously unidentified areas of Pax2 expression in the developing mouse forebrain. Further, we show that Pax2 expression is maintained in the septum of *Pax6*^*Sey*/*Sey *^mutants, which are known to have an enlarged septum. This study highlights the value of Pax2 as a novel marker of forebrain development.

## Results

### Domains of Pax2 expression in the developing mouse forebrain

**Figure 1:**
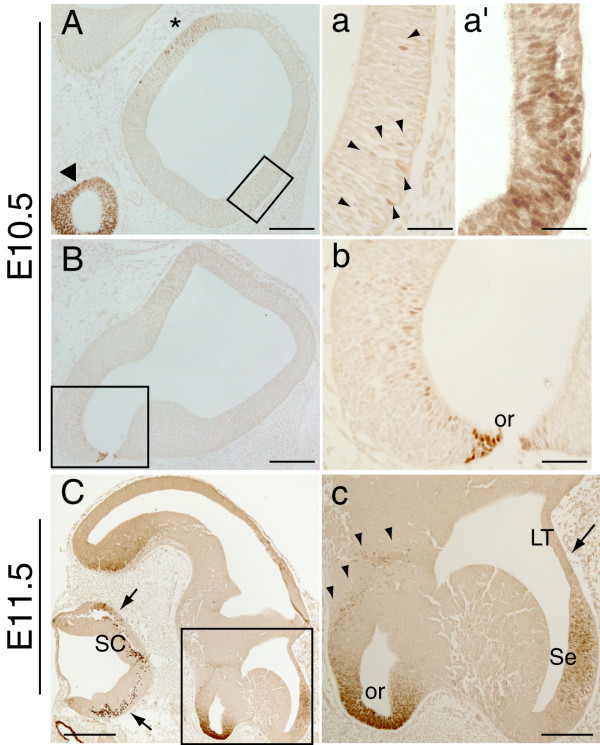
**Pax2 protein expression in the mouse forebrain at E10.5 and E11.5**. **A-B**, **a-b**: E10.5 sagittal sections showing Pax2 expression in the developing forebrain. In the ventral telencephalon, a few Pax2-positive cells can be detected in lateral sections (arrowheads in **a**) and become more abundant in more medial sections (**a'**). A few Pax2-positive cells are detected in the hypothalamus, close to the strongly Pax2-positive optic recess (or) area (**B, b**). The previously described staining in the future eminentia thalami of the diencephalon (asterisk in **A**) and the optic cup (arrowhead in **A**) are also shown. **C**, **c**: E11.5 sagittal sections revealing strong Pax2 expression in the developing septum (Se). No Pax2 expression is detected in the neighbouring lamina terminalis (LT) (arrow in **c**) at this developmental stage. Pax2-immunopositive cells are also found in the anterior hypothalamus (arrowheads in **c**) and may originate from the ventral neuroepithelium of the optic recess area (or). Expression in the eminentia thalami is not shown. Note the Pax2 expression in the spinal cord (SC) (arrows in **C**). **a, b **and **c **are high power images of the boxed areas in **A, B **and **C **respectively. **a' **is a high power image of a sagittal section at a more medial level than that depicted in **a**. Scale bars: **C**, 400 μm; **A**, **B**, **c**, 200 μm; **a**, **a'**, **b**, 50 μm.

We examined Pax2 expression in the early forebrain using immunohistochemistry on sagittal sections of E10.5 and E11.5 embryos (Fig. 1). At E10.5, a few Pax2-immunopositive cells were detected within the ventral telencephalon in lateral sagittal sections (Fig. 1A, a-arrowheads), with the staining becoming more intense in mid-sagittal sections (Fig. 1a'). In addition, a small population of Pax2 immunopositive cells was detected in the neuroepithelium of the anterior hypothalamus, adjacent to the optic recess area (Fig. 1B,b). At E11.5, strong Pax2 expression was found in the developing septum (Fig. 1C,c) with no Pax2-immunopositive cells detected in the neighbouring lamina terminalis (arrow in Fig. 1c). As in E10.5 embryos, a number of Pax2-positive cells were found close to the base of the hypothalamus but also in more dorsal areas of the anterior hypothalamus (Fig. 1C,c). At both E10.5 and E11.5, Pax2 expression was found in previously described regions such as the ventral neuroepithelium of the optic recess (Fig. 1B,b,C,c) [5,7,9,10], the optic cup (arrowhead in Fig. 1A and not shown) [5,7,9,10] and near the diencephalic-telencephalic boundary (asterisk in Fig. 1A and not shown), which will give rise to the eminentia thalami [21].

**Figure 2:**
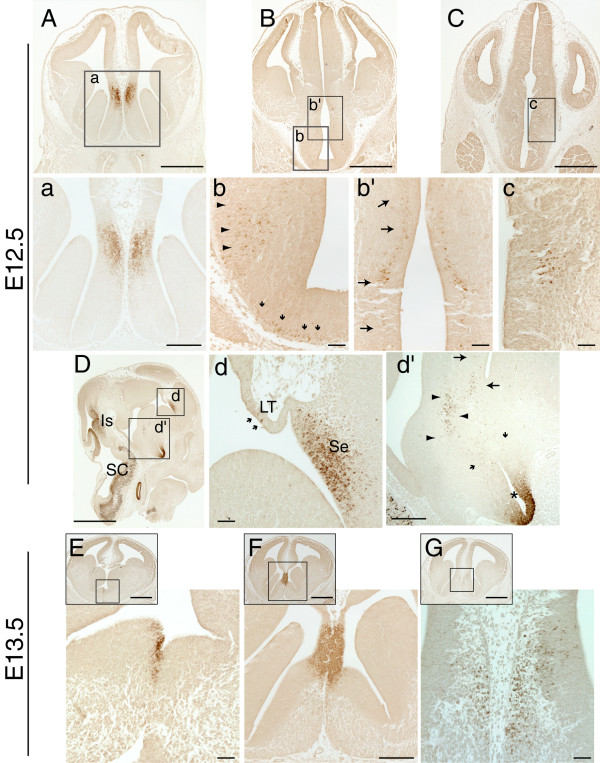
**Pax2 protein expression in the mouse forebrain at E12.5 and E13.5**. **A-C**: Low power views of coronal E12.5 forebrain sections immunoreacted with Pax2; the boxed areas are shown at higher magnification in panels **a-c**. **D**: Low power view of a sagittal E12.5 section immunoreacted with Pax2. Note the strong expression of Pax2 in the isthmic region (Is) and the spinal cord (SC), in accordance with previous reports. **d**, **d' **higher magnification of the boxed areas in **D**. **A**, **a**, **d**: Strong Pax2 expression is detected in the septum (Se) of the basal forebrain, mainly in the septal neuroepithelium. A few Pax2-positive cells are detected in the lamina terminalis (LT) (arrows in **d**). **b-c**, **d'**: Pax2 is detected in groups of cells found in the lateral hypothalamic area (arrowheads in **b **and **d'**) and in the anterior hypothalamic neuroepithelium (arrows in **b' **and **d'**, **c**). These cell populations might originate from cells located at the base of the hypothalamus (small arrows in **b **and **d'**), Pax2 expression can also be seen in the ventral neuroepithelium of the optic recess (asterisk in **d'**), as previously described. **E-G**: Low and high power images of E13.5 coronal sections reveal strong Pax2 expression in the septum, mainly in the neuroepithelium (**F**). A few immunopositive cells are found in the differentiating layer of the septum (**G**). The boxed areas in the inset panels in **E-G **indicate the areas shown in the respective high power images. **A-C **and **E-G **are sections from the same specimens respectively. Scale bars: **D**, **E-G**-insets, 1000 μm; **A-C**, 500 μm; **a**, **d'**, **F**-high power, 200 μm; **b-c**, **d**, **E**, **G**-high power, 50 μm.

Pax2 protein expression was then examined at E12.5, on coronal and sagittal sections along the caudo-rostral axis of the developing mouse forebrain (Fig. 2). The strongest expression domain of Pax2 was detected in the telencephalon. The Pax2 antibody labelled most cells of the septal neuroepithelium, located in close proximity to the dorso-medial telencephalon (Fig. 2A,a). This strong and characteristic Pax2 expression in the septum was also observed in sagittal sections, in the region where the lamina terminalis joins the septum (Fig. 2D,d). Only a few Pax2-immunopositive cells were detected within the neighbouring lamina terminalis (arrows in Fig. 2d).

Specific Pax2 expression was also detected in small clusters of cells in regions of the hypothalamus. The Pax2 antibody labelled a group of cells located at the lateral hypothalamic area (Fig. 2B, arrowheads in b), and a narrow strip of cells parallel to the anterior hypothalamic ventricular zone (Fig. 2B, arrows in b'). A few Pax2-immunopositive cells were also detected along the base of the hypothalamus, excluding the midline region (Fig. 2b, arrows). These groups of Pax2-positive cells can also be distinguished in a sagittal plane (Fig. 2D,d'). The cell populations indicated by the arrowheads and small arrows (Fig. 2d'), located just above the optic recess area, correspond to the respective populations indicated in Fig. 2b. The Pax2 positive cells located close to the third ventricle, indicated with large arrows in Fig. 2d', correspond to those shown in Fig. 2b'. In coronal sections of the caudal forebrain, specific Pax2 expression was also detected in a small cluster of cells resembling a nucleus in the neuroepithelium of the anterior hypothalamus (Fig. 2C,c). In accordance with previous reports, Pax2 expression was also detected in the ventral neuroepithelium of the optic recess area at the base of the hypothalamus (Fig. 2d', asterisk), retina (not shown) [5,7,9,10] and eminentia thalami at the level depicted in Fig. 2B (not shown) [21].

**Figure 3:**
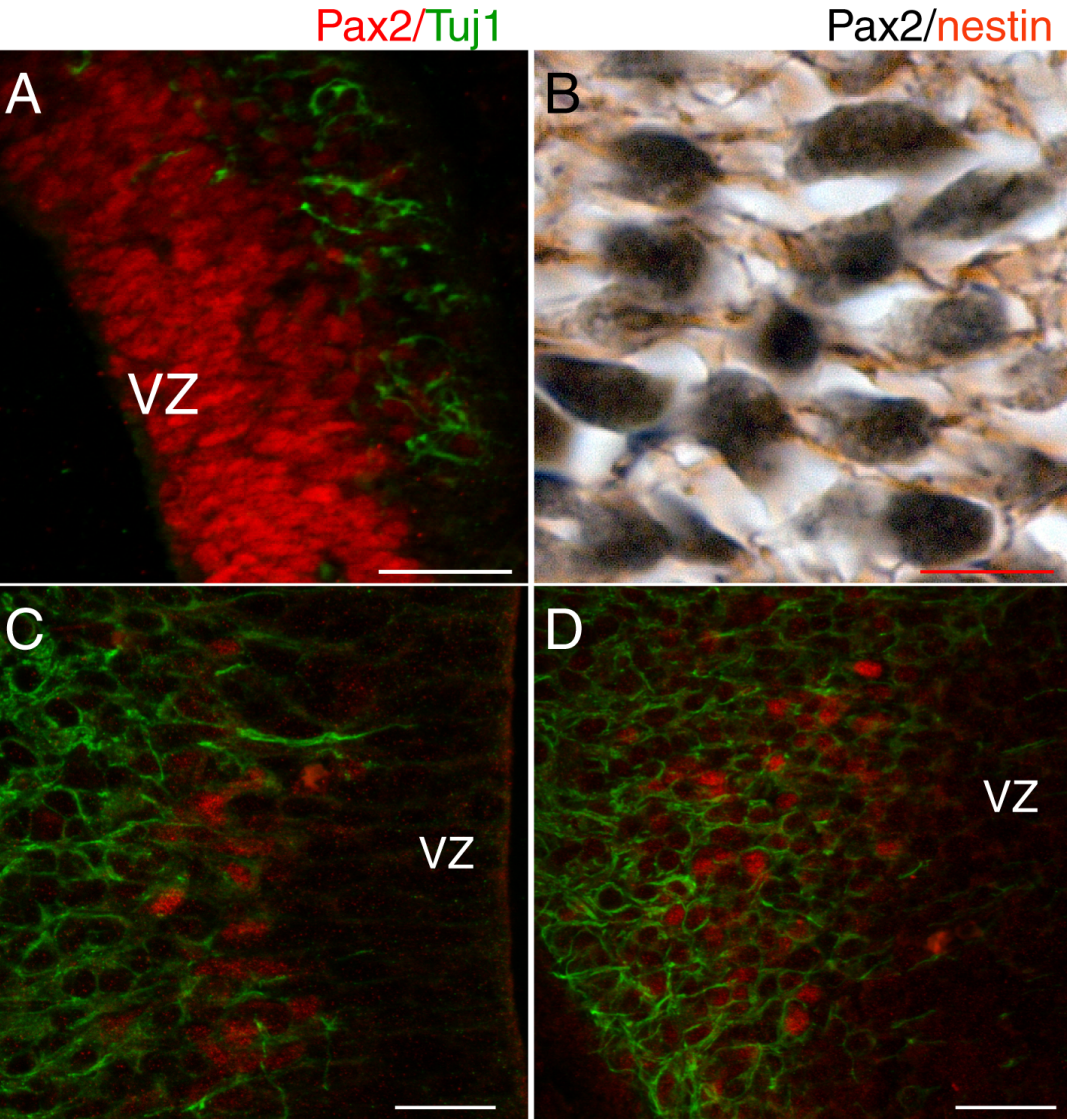
**Pax2 is primarily expressed in neural progenitors in the septum and in early differentiated neurons in the hypothalamus**. **A**: Double immunofluorescence with Pax2 (red) and β-tubulin III (Tuj1) (green) on a sagittal E12.5 telencephalic section shows that only a minority of Pax2-positive cells in the septum also express Tuj1, an early marker of differentiated neurons. This is further confirmed with double immunohistochemistry with Pax2 (black) and nestin (brown) (**B**), revealing that these Pax2-positive cells have nestin-positive filaments. Panels (**A**) and (**B**) show correspond to high power images taken within the region depicted in **Fig. 2D, d**. In the hypothalamus (**C, D**), most Pax2-positive cells express the early neural marker β-tubulin III (green), showing that they correspond to early generated neurons. Pax2-positive cells in (**C**) correspond to those shown in **Fig. 2b'**, and those in (**D**) correspond to the cells indicated by arrowheads in **Fig. 2b**. The position of the ventricular zone (VZ) is indicated in sections **A, C **and **D**. Scale bars: **A**, 20 μm; **B-D**, 5 μm.

Double immunofluorescence with Pax2 and β-tubulin III (Tuj1), a marker of early neural differentiation found in neurites, revealed that a very low proportion of septal cells labelled with Pax2 co-expressed β-tubulin III (Fig 3A), suggesting that the majority of the Pax2-positive cells in this region are neural precursors. This was confirmed by double immunohistochemistry with Pax2 (black, nuclear staining) and nestin (brown, filament staining), an intermediate filament protein found in radial glia, which showed that the β-tubulin III-negative/Pax2-positive cells in the septum express nestin (Fig. 3B). In the hypothalamus, most of the Pax2-positive cells found close to the ventricle (panels 2b' and 2c) are also positive for β-tubulin III, showing that these cells are newly formed neurons (Fig. 3C). The Pax2-positive population indicated by arrowheads in panels 2b and 2d' also expressed β-tubulin III, even more extensively than the other hypothalamic Pax2-positive cells (Fig. 3D). Finally, the Pax2-positive cells located close to the hypothalamic ventral midline (small arrows in panels 2b and 2d') did not express β-tubulin III, but were positive for nestin, indicating that they correspond to neural progenitors (data not shown).

At E13.5, Pax2 was detected in cells located at the septal midline (Fig. 2E). Expression was strong and specifically confined to the septal neuroepithelium, at the point where the septum joins with the future hippocampus via the lamina terminalis (Fig. 2F). At more rostral telencephalic levels, a small number of Pax2-immunopositive cells were found in the differentiating field of the septum (Fig. 2G).

Pax2 expression in the hypothalamus at this age was similar to that seen at E12.5, with the exception that the immunopositive cells in the lateral hypothalamic area and the base of the hypothalamus shown in Fig. 2b, were no longer detectable (data not shown). No Pax2-immunopositive cells were found in the eminentia thalami after E13.5 (not shown).

**Figure 4:**
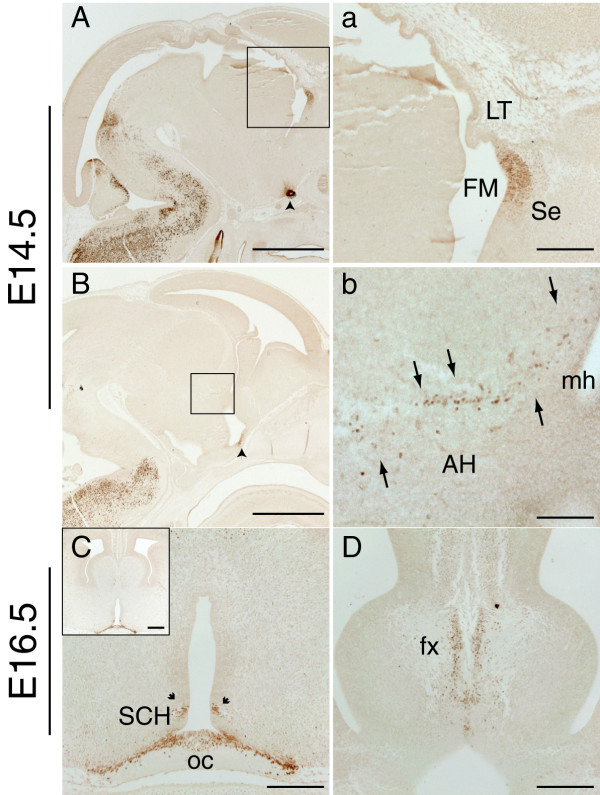
**Pax2 protein expression in the mouse forebrain at E14.5 and E16.5**. **A**, **a**: Low and high power images of an E14.5 sagittal section showing strong Pax2 staining in the neuroepithelium of the septum (Se) adjacent to the foramen of Monro (FM) in an E14.5 sagittal section. This intense Pax2 staining is observed at the level where the lamina terminalis (LT) joins the septum. **a **shows a higher magnification of the boxed area in panel **A**. **B**, **b**: In more lateral sagittal sections Pax2 expression is found in a cell population (arrows) within the anterior hypothalamus (AH) reaching the medial horn (mh) of the lateral ventricle. **b **shows a higher magnification of the boxed area in panel **B**. **C**: E16.5 coronal section showing Pax2 expression around the optic chiasm (oc) region, in the optic stalk epithelium and in a few cells of the hypothalamic neuroepithelium next to the suprachiasmatic nucleus (SCH) (arrows). **D**: E16.5 coronal section showing Pax2 expression in the differentiating layer of the medial septum surrounded by the axonal bundles of the fornix (fx). Scale bars: **A**, **B**, 1000 μm; **a**, **C**, **D**, 200 μm; **C**-inset, 250 μm; **b**, 100 μm.

At E14.5, coronal telencephalic sections revealed similar Pax2 expression to that described at E13.5 (data not shown). As for the earlier ages examined, Pax2 expression was mainly confined to the septum, in the neuroepithelium adjacent to the lamina terminalis, as depicted in the mid-sagittal section in Fig. 4A and 4a. In the E14.5 hypothalamus, Pax2 expression was limited to a very narrow band of cells, localized in the differentiating field of the anterior hypothalamus and reaching the medial horn of the lateral ventricle (Fig. 4B, arrows in b). We could not detect these immunopositive cells in the coronal plane, probably because of their narrow field of expression. Expression was also found at the optic recess area as previously described [9], (arrowhead in Fig. 4A,B).

By E16.5, the main Pax2 expression domain in the developing septum was located in the differentiating field of the medial septum. It was mainly found in a stripe-like cell arrangement, parallel to the midline and surrounded by the fibre bundles of the fornix (Fig. 4D). At this developmental stage, the only Pax2 expression detected in the hypothalamus was in the optic stalk epithelium, just above the optic chiasm region (Fig. 4C), in a few cells of the hypothalamic neuroepithelium, adjacent to the suprachiasmatic nucleus (arrowheads in Fig. 4C) and in a few scattered cells in the medial preoptic nucleus.

**Figure 5:**
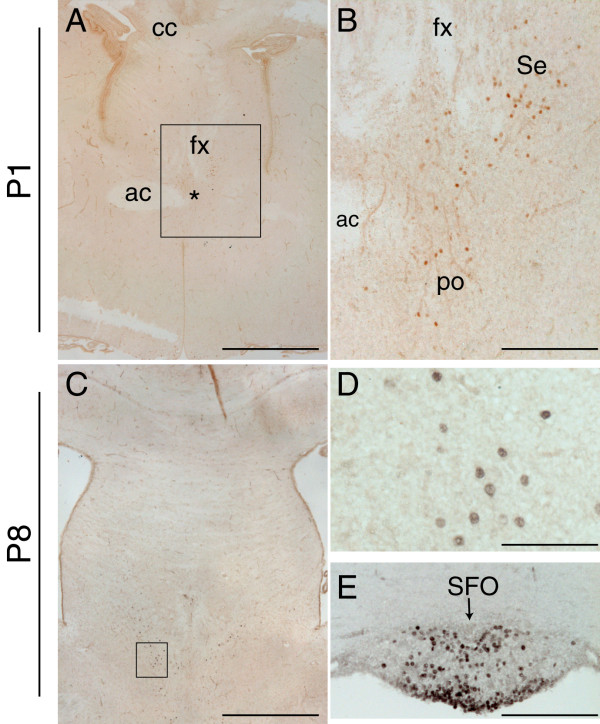
**Pax2 protein expression in the mouse forebrain during early postnatal development**. **A-E**: Coronal forebrain sections immunostained for Pax2 at P1 (**A-B**) and at P8 (**C-E**), showing the presence of few disperse Pax2-positive cells in the septal area (Se) (**A-C**, high power in **D**), the medial preoptic nucleus (po) (asterisk in **A **and **B**), and the subfornical organ (SFO) (arrow in **E**). The fibre tracts are indicated in **A **and **B **for orientation purposes (ac, anterior commissure; cc, corpus callosum; fx, fornix). The boxed areas in **A **and **C **delineate the high power images depicted in B and D respectively. Scale bars: **A**, **C**, 100 μm; **B**, **E**, 25 μm; **D**, 12.5 μm.

During early postnatal development only a few Pax2-positive cells were detected in the forebrain. At postnatal day (P) 1, Pax2 immunopositive cells were detected scattered in the most caudal sections of the septal area, at the level where the fornix is found in proximity to the anterior commissure (Fig. 5A). As at E16.5, a few cells were found close to the fornix, in the medial septal area, as well as in the border zone between the medial and lateral septum (Fig. 5A,B). Pax2 also labelled the medial preoptic nucleus of the hypothalamus (Fig. 5B). At P8, a similar expression pattern was observed (Fig. 5C, D). In addition, Pax2 was detected in the subfornical organ (Fig. 5E), one of the circumventricular organs of the brain involved in fluid balance [25]. Pax2 expression was no longer detected at P15 (not shown).

### Expression of Pax2 in the septum in *Pax6*^*Sey*/*Sey *^mutants

**Figure 6:**
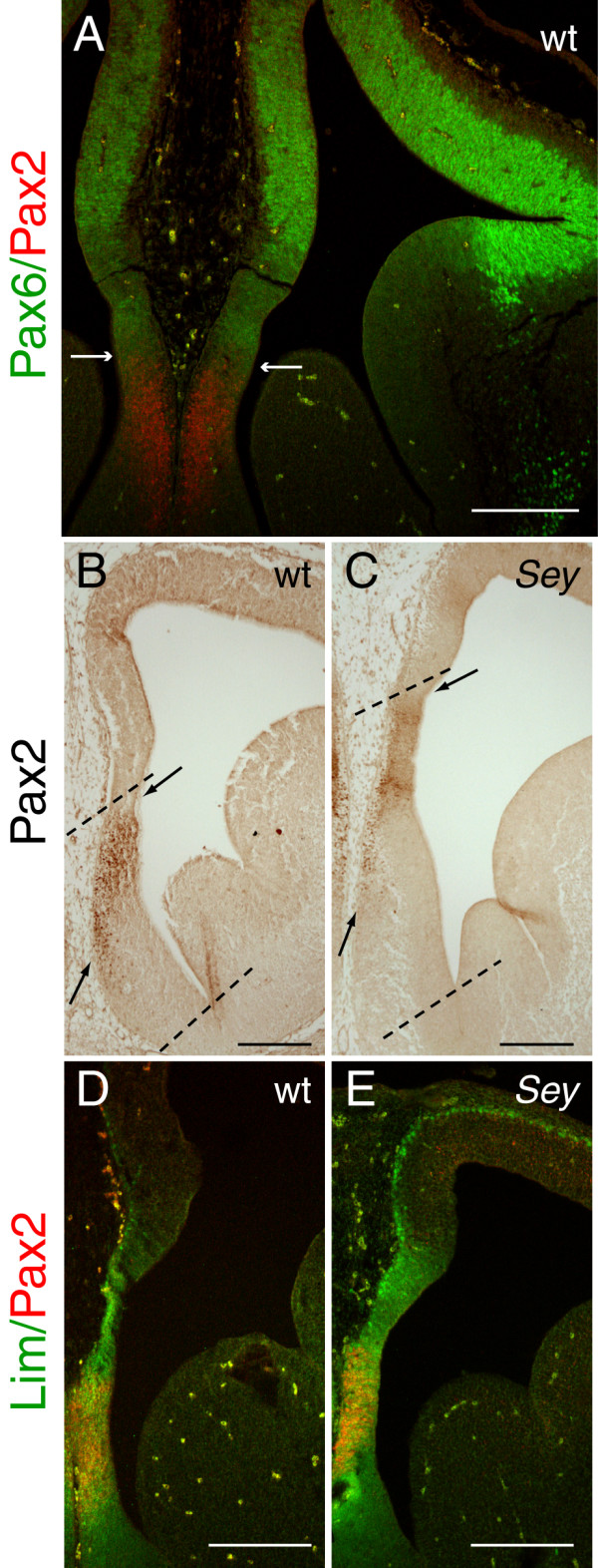
**The Pax2 expression domain in the telencephalic septum is located in a more dorsal position in the *Pax6*^*Sey*/*Sey *^mutant than in wild type**. Pax2 expression in the telencephalic septum in E12.5 wild type (wt) (**A, B, D**) and *Pax6*^*Sey*/*Sey *^mutant (*Sey*) (**C, E**) coronal sections. **A**: Double immunofluorescence with Pax2 (red) and Pax6 (green) reveals that the two proteins are expressed in non-overlapping, mutually exclusive domains. The arrows in **A **indicate the ventral and dorsal limits of Pax6 and Pax2 expression respectively. **B-C**: Pax2 expression in wt and *Sey *embryos, showing that the Pax2 expression domain is shifted dorsally in the *Sey *mutant (**C**) compared to wt (**B**). The septum and the Pax2 expression domain are indicated by dashed lines and arrows respectively. **D-E**: Double immunofluorescence with Pax2 (red) and Lim1/2 (green) in the septum shows that Pax2 expression is found within the Lim-positive domain in both wild types (**D**) and *Sey *mutants (**E**), suggesting that the shifted Pax2 expression domain in the *Sey *mutant is still within the limits of the ventral telencephalon. Note that panels B and C correspond to slightly more rostral sections than those shown in D and E respectively. Scale bars: **A**, **B**, **C**, 200 μm; **D**, **E**, 100 μm.

To validate Pax2 as a marker of the septal neuroepithelium, we used the *Pax6*^*Sey*/*Sey *^mutant, which has been shown to have an enlarged septum [15]. Using double immunofluoresence, we first examined expression of Pax2 and Pax6 in E12.5 wild type embryos. In rostral telencephalic sections, Pax6 was strongly expressed in the dorsal, lateral and ventral pallium, with its most ventral expression domain expanding into the lateral ganglionic eminence, just below the pallial-subpallial boundary (Fig. 6A), as previously described [11,14,24]. Pax6 was expressed at lower levels in the medial pallium but did not overlap with the expression domain of Pax2 in the septal neuroepithelium (Fig. 6A, arrows).

Using *Pax6*^*Sey*/*Sey *^mutant embryos, we examined whether loss of Pax6 affects Pax2 expression in the septum. No gross changes in the extent of the main Pax2 expression domain were observed, although the intensity of Pax2 staining appeared reduced in *Pax6*^*Sey*/*Sey *^mutants compared to the wild type (area between arrows in Fig. 6B,C). In addition, Pax2 expression was observed in a more dorsal area compared to wild type, revealing a larger septum in the mutant (compare areas between dotted lines in Fig. 6B and 6C), in accordance with previous published data [15]. This result was consistent in all mutants examined (n = 6). Double immunofluorescence with antibodies for Pax2 and the septal marker Lim1 (also known as Lhx1), using an antibody that detects both Lim1 and Lim2 (Lim1/2) [26,27], showed that in both wild types and *Pax6*^*Sey*/*Sey *^mutants, Pax2 expression was confined within the Lim1/2 positive domain (Fig. 6D,E). This shows that the shifted area of Pax2 expression observed in the *Pax6*^*Sey*/*Sey *^mutant most likely corresponds to ventral telencephalic tissue, and not to ectopic Pax2 expression in the dorsal telencephalon.

### Pax2-positive cells in the early postnatal septum do not express markers of GABA-ergic or cholinergic neurons

To gain further insight into the neurochemical properties of the differentiated Pax2 cells detected in the septum, we examined co-expression of Pax2 and markers of GABA-ergic and cholinergic neurons, the principal neuronal types of this structure [28-30].

**Figure 7:**
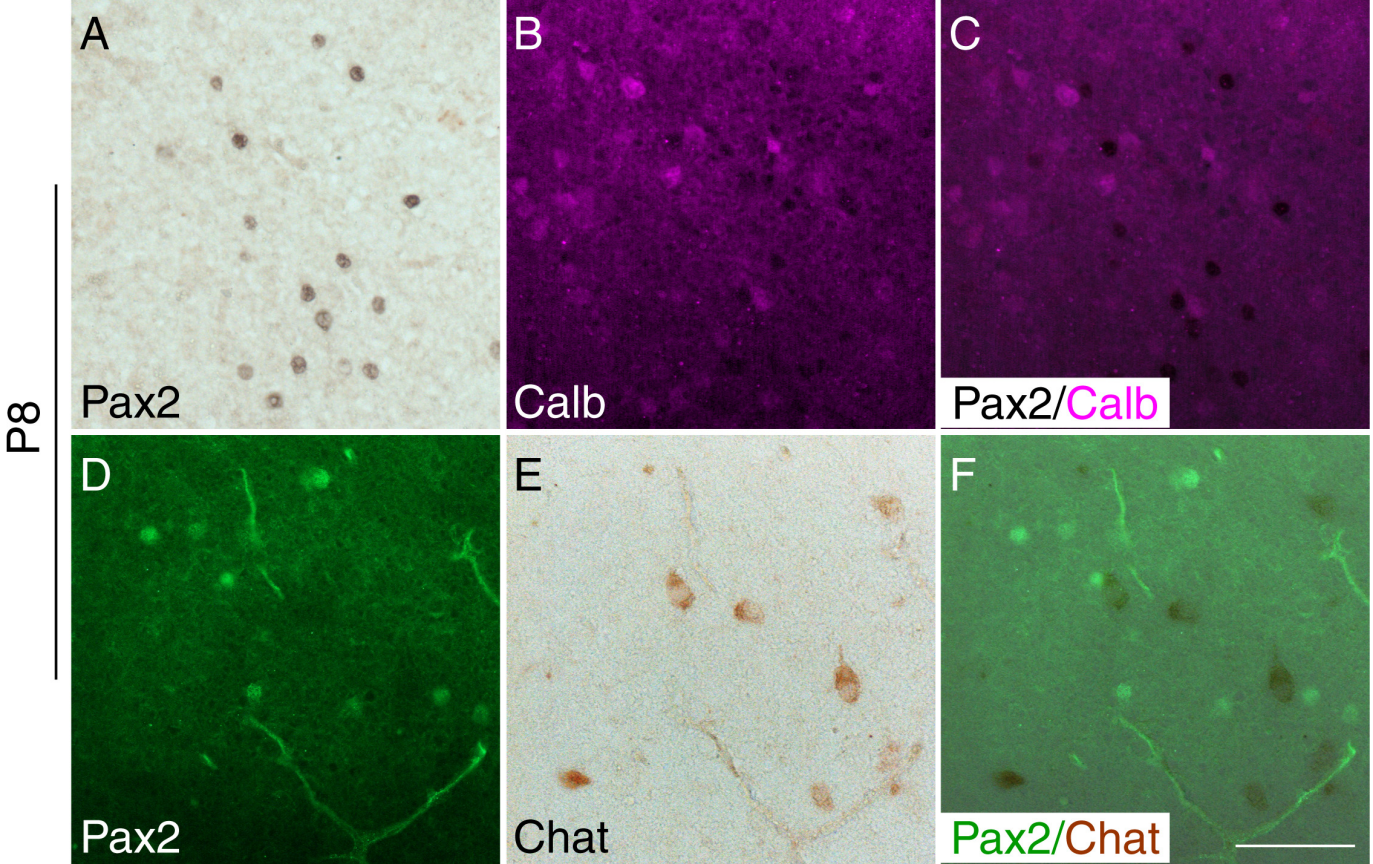
**Pax2 expression in the medial septum does not co-localise with that of calbindin and choline acetyltransferase (Chat)**. **A-C**: Double immunohistochemistry with Pax2 (black) (**A**) and calbindin (magenta) (**B**) on P8 coronal sections reveal the presence of both calbindin-positive and Pax2-positive neurons in the septal area, but not co-expression of these proteins (**C**). Similarly (**D-F**), septal neurons that express Pax2 (green) (**D**) and Chat (brown) (**E**), do not co-express these proteins (**F**). The panels are high powers of the level depicted in **Fig. 5C**. Scale bar for all panels, 10 μm.

To examine whether the Pax2-positive cells detected postnatally might be GABA-ergic we performed double immunostaining experiments with Pax2 and calbindin, a calcium-binding protein that has been shown to label a large population of GABA-ergic somatospiny neurons in the adult septum [31,32]. A large number of neurons were labelled with an antibody for calbindin in the postnatal septum (Fig. 7B). However, Pax2-positive cells did not colocalize with calbindin, neither at P1 (not shown) nor at P8 (Fig. 7A–C). To examine whether the Pax2 immunolabelled neurons might be cholinergic, we performed double immunostaining experiments with appropriate markers. At P1, we examined co-expression of Pax2 and Islet1, a protein that has been shown to label some populations of cholinergic septal neurons [33,34]. No co-localisation of these two proteins was detected (not shown). Choline acetyltransferase (Chat), the acetylcholine-synthesizing enzyme in cholinergic neurons, can not be detected clearly in septal neurons by means of immunohistochemistry before P8 [28,30]. We examined co-localization of Chat and Pax2 at P8 by means of double immunostaining (immunohistochemistry followed by immunofluorescence). As shown in Fig. 7, at P8 a few cells in the septum start expressing Chat but do not express Pax2 (7D–F).

## Discussion

Specific markers expressed in different regions of the developing nervous system are widely used as tools to study neural development. Here, the expression of Pax2, a well-studied marker of the isthmus, spinal cord and developing eye, has been re-examined, using a polyclonal antibody, and novel areas of Pax2 protein expression have been identified in the ventral telencephalic septum and the developing hypothalamus. The polyclonal antibody used in the present study recognizes the same epitope described by Dressler and Douglass (1992) and has been previously used by several groups to characterize Pax2 protein distribution [2,7,9]. In this study, the use of paraffin sections subjected to microwaving for antigen retrieval, in contrast to the cryostat sections used previously, may have allowed the identification of the previously undescribed domains of Pax2 expression.

Pax2 staining in the hypothalamus is first detected at E10.5 and comprises a few cells in the ventricular zone, dorsal to the optic recess. By E11.5, Pax2 positive cells are found in a region that extends from the area of the optic recess to the lateral ventricle that will give rise to the anterior hypothalamus. Comparison of the distribution of Pax2 positive cells in this region at E12.5 and E10.5 suggests that they might follow a migratory path towards dorsal regions of the anterior hypothalamus. By E14.5, fewer Pax2-positive cells are present than at E12.5, and these are restricted to the dorsal anterior hypothalamus. Pax2 is expressed in the optic stalk, a structure that joins the optic cup to the brain, and that ends at the base of the hypothalamus [5,7,9,10]. It is therefore possible that the small population of Pax2/β tubulin III-positive cells in the developing anterior hypothalamus described here, may arise from the optic stalk and collaborate with other cellular hypothalamic cues in guiding the trajectory of the optic nerve.

Pax2 is also expressed in the eminentia thalami, a transient developmental structure of unknown function [35] that joins the ventral diencephalon to the telencephalon [22-24]. Pax2 expression in the eminentia thalami is first detected at E10.5 at the dorsal border between the diencephalon and telencephalon [21], and this staining can not be detected after E13.5. The Pax2-positive cells in the eminentia thalami do not express β-tubulin III, indicating that they are most likely to be neuronal precursors. The early appearance of these cells at the diencephalic-telencephalic boundary (this study and [21]) suggests that they might be important for the formation of this boundary.

Consistent, high levels of Pax2 expression were also observed in the septum of the basal forebrain. Pax2 is first expressed at E10.5 by a small number of cells located in the ventral telencephalon. It seems likely that these cells give rise to the Pax2-positive population observed in the septal area one day later. By E12.5, septal Pax2 expression although strong, is confined to the septal neuroepithelium, mainly at levels proximal to the lamina terminalis. Only a few Pax2-positive cells are observed in the differentiating layer of the septum. Septal expression is also observed at later developmental stages, but by E16.5 it is downregulated and becomes restricted to a small population of differentiated cells in the medial septum. During septal development in rodents, cells migrate from the lateral ventricle towards the midline. The medial nucleus is one of the first nuclei formed in the septum [36-38]. Therefore, it is possible that the sparse Pax2-positive cells observed at E16.5 correspond to the Pax2-positive cells observed in the differentiating field of the septum between E12.5 and E14.5 (Fig. 3A, 2G and not shown).

Pax2 is still expressed by a few cells located in the medial and lateral septal areas during postnatal development and it is not detected after P15. The majority of neurons in the medial and lateral septum are cholinergic or GABA-ergic [28,30,31]. Mature cholinergic neurons express the enzyme choline acetyltransferase (Chat) [39,40], whose expression in the septum becomes detectable at around P8 [28,30]. At this age, Pax2 expressing neurons are still found in the septal region but do not co-express Chat, suggesting that these cells might not be of the cholinergic type. However, it is also possible that the Pax2 immunopositive cells might develop into cholinergic neurons at later time points when Chat expression has increased and Pax2 expression has been turned off. Similarly, calbindin is expressed by a large number of somatospiny GABA-ergic neurons in the adult septum [31,32] and it is present in the postnatal septal area, but it does not co-localize with Pax2, indicating that the Pax2 septal neurons are not of this particular GABA-ergic type. As there many different types of GABA-ergic neurons in the septum [41], it is possible that the Pax2-positive cells might develop into a different type, such as the septohippocampal projection neurons, a prominent GABA-ergic population comprised of parvalbumin-positive cells [28,42,43]. Although a few of these cells are first detected at around P8 [28,30], when Pax2 expression is still detectable in the septum, they become clearly visible after P15, when Pax2 expressing cells are no longer present in the septum. Again, this expression pattern precludes us from being able to draw conclusions about the specific neuronal type of these cells based solely on immunostaining techniques. Cell fate experiments using a Pax2-cre mouse strain and an appropriate cre reporter strain would allow us to address which type of neurotransmitter fate and properties the Pax2-positive septal neurons adopt.

In the developing eye, there is a sharp boundary between the domains of Pax2 expression in the optic stalk and Pax6 expression in the optic cup, [18,44,45]. Mutual cross-repressive interactions between Pax2 and Pax6 are essential for formation of this boundary [45]. Here we show that Pax2 and Pax6 are expressed in neighbouring, non-overlapping domains in the rostral telencephalon, reminiscent of the pattern observed in the developing eye [18,45]. Given this expression pattern, and the fact that the septum is enlarged in *Pax6*^*Sey*/*Sey *^mutants [15], we hypothesised that the Pax2 septal expression domain might be expanded in this mutant. However, we found no increase in the extent or intensity of Pax2 expression in this region in *Pax6*^*Sey*/*Sey *^mutants. Nevertheless, the Pax2 expression domain was found at a more dorsal position than in the wild type, consistent with the previously described size increase of the septum in the *Pax6*^*Sey*/*Sey *^mutant [15]. The Pax2 expression domain still lies within the ventral telencephalon, as shown by co-expression of the septal marker Lim1 [26,27].

There are several mouse models with different types of mutations in the *Pax2 *locus, including the Krd mice (Krd/+), a mutant with a chromosomal deletion that includes this locus [9,46,47], *Pax2*^-/- ^null mutants [10,48], and *Pax2*^1*Neu *^mice with a frameshift mutation in *Pax2 *[49]. All of these mutants display defects in kidney formation, optic nerve trajectory, and inner ear patterning, consistent with previously identified expression domains of *Pax2 *transcript [10,48-50]. However, defects in the midbrain-hindbrain region range from complete loss of the posterior mesencephalon and cerebellum in the *Pax2*^1*Neu *^mouse [49], to no phenotypic alteration in the *Pax2*^-/- ^mutant [10,51], possibly as a consequence of differences in genetic background [10]. It would be of interest to study the telencephalic septum in these different mutants, to identify any possible alterations due to loss of Pax2 expression in this region.

## Conclusion

Pax2 is expressed in the anterior hypothalamus, eminentia thalami and telencephalic septum in the developing mouse forebrain, in neuronal progenitors and early born neurons. Between E11.5 and E14.5, it is strongly expressed in the septal neuroepithelium, at a level close to the lamina terminalis and it is no longer detectable by P15. Further, the absence of functional Pax6 does not cause gross alterations of Pax2 expression in the septum. Thus Pax2 represents an ideal marker for the study of the developing septum.

## Methods

### Mice

Animal care was in accordance with institutional guidelines and UK Home Office regulations. The day the vaginal plug was detected was considered E0.5. Wild type mice on a CBA genetic background were used for the Pax2 expression pattern analysis. *Pax6*^*Sey*/+ ^heterozygotes, kept on a mixed CD1-Swiss genetic background, were intercrossed to generate homozygous null embryos. These were identified by the absence of eyes, as previously described [13]. Wild type mice from the same genetic background were collected for comparison by intercrossing wild types.

#### Histology

Embryos were harvested between E10.5 and E16.5 and pups between P1 and P15. Whole embryos or heads of P1 were immersion fixed in 4% paraformaldehyde in 0.1 M phosphate buffer overnight at 4°C. P7 and P15 pups were anesthetized with Avertin and perfused through the heart with fixative, followed by tissue dissection and overnight incubation in fresh fix. Embryonic and postnatal tissue were all processed following standard conditions [21], embedded in paraffin and cut in serial 10 μm sections (embryonic tissue) or 12.5 μm (postnatal brains) at a coronal or sagittal plane. At least two wild type embryonic heads or postnatal brains for each age were used, and six *Pax6*^*Sey*/*Sey *^mutants with the corresponding wild types were examined with each marker.

#### Immunohistochemistry and Immunofluorescence

These were performed according to standard protocols. Antigen retrieval was achieved by microwaving sections at full power in 10 mM citrate buffer, pH 6.0 for four times, 5 min each. Primary antibodies were rabbit anti-Pax2 (Covance, 1:200), rabbit anti-calbindin (Swant, 1:500), monoclonal mouse anti-Pax6, Lim1/2 (DSHB, both 1:100) and nestin (DSHB, 1:50), and goat anti-choline acetyltransferase (Millipore – AB144P, 1:50). Secondary non-fluorescence antibodies were biotylinated anti-rabbit IgG (Dako, 1:200), biotylinated anti-mouse IgG (Dako, 1:200), biotylinated anti-goat IgG (Dako, 1:100),

For single immunohistochemistry experiments the dark brown signal was revealed after incubation with the ABC kit (Vector), followed by standard diaminobenzidin (DAB, Sigma) and hydrogen peroxide incubation. For the double immunohistochemistry experiments, nuclear staining was first detected using a DAB-nickel detection kit (Vector) resulting in a grey/black staining. Sections were then incubated with the second antibody and appropriate secondary and the dark brown filamentous signal was revealed after incubation with the ABC kit (Vector), followed by a DAB reaction using the DAB detection kit (Vector). For the double immunofluorescence experiments, Pax6 or Lim1/2 signal were amplified with a biotinylated anti-mouse IgG antibody and signal was revealed after incubating with streptavidin conjugated to Alexa fluor 488 dye (Invitrogen, 1:200). Monoclonal anti-β tubulin isotype III (Sigma – clone SDL.3D10, 1:400) was detected using as secondary antibody an anti-mouse IgG conjugated to Alexa fluor 488 dye (Invitrogen, 1:200). For double immunohistochemistry followed by immunofluorescence, after detection of the first antibody with DAB or DAB-Nickel immunohistochemistry as described above, sections were incubated with a second antibody which was detected by means of immunofluorescence. Polyclonal Pax2 and calbindin were detected using as secondary antibodies an anti-rabbit IgG conjugated to Alexa fluor 488 dye and an anti-rabbit IgG conjugated to Alexa fluor 568 dye, respectively (Invitrogen, 1:200). Appropriate controls were used in all cases by incubating some sections with all but the primary antibodies. No immunostaining occurred under these conditions.

### Microscopy

A Leica microscope connected to a Leica DFC 480 digital camera was used to capture images of DAB and immunofluorescent labelled sections. Confocal images were captured with a Leica TCS NT confocal microscope.

## Abbreviations

E: embryonic day; P: postnatal day; Chat: choline acetyltransferase.

## Authors' contributions

VF designed and carried out the experiments, analysed the results and wrote the manuscript. DJP and JOM participated in the analysis and writing of the manuscript. All authors read and approved the final manuscript.
